# Change of Renal Parenchymal Width in Patients with Unilateral Ureteral Stent: A Bicenter Retrospective Study

**DOI:** 10.1155/2017/1653184

**Published:** 2017-06-01

**Authors:** Hee Youn Kim, Seung-Ju Lee, Jin Bong Choi, Je Mo Yoo, Joon Ho Lee, Dong Sup Lee

**Affiliations:** ^1^Department of Urology, St. Vincent's Hospital, College of Medicine, The Catholic University of Korea, Suwon, Republic of Korea; ^2^Department of Urology, Seoul St. Mary's Hospital, College of Medicine, The Catholic University of Korea, Seoul, Republic of Korea

## Abstract

**Purpose:**

To determine whether kidney sizes were changed after ureteral stents were instilled, and if so, what parameters were significant.

**Methods:**

Parenchymal width (PW) of 98 patients with unilateral ureteral stents was measured from the coronal view of CT scans for both stented and unstented contralateral kidney. The mean PW and % change of mean PW were calculated before stenting and at the time of last stent change. Estimated glomerular filtrate rate (eGFR) was recorded as well.

**Results:**

The mean duration of ureteral stent indwelled was 15.6 ± 10.2 (mean ± SD) months. The change of mean PW of stented kidneys and unstented contralateral kidneys was −16.9 ± 16.4 (mean ± SD)% and 3.6 ± 10.7%, respectively. eGFR before and at the time of the last stent change did not show significant difference (*p* = 0.294). Duration of ureteral stent indwelled was found to be inversely related to the % change of mean PW (Spearman's correlation coefficient = −0.291, *p* < 0.001).

**Conclusions:**

For unilateral ureteral obstruction, kidney size was decreased over time in spite of indwelling ureteral stent. This finding can be overlooked by clinicians due to compensatory growth of contralateral kidney and resultant normal eGFR.

## 1. Introduction

 Ureteral stents are widely used for treating benign or malignant obstruction of ureter caused by various pathologies. One of the main rationales for inserting ureteral stents is in their capacity in improving and preserving renal function of the obstructed kidney by adequately draining urine. While previous studies have demonstrated the high success rate of ureteral stents for managing ureteral obstructions [1–3], to the best of our knowledge, no studies have focused on selective renal function of stented kidney itself except in the context of measuring laboratory values for renal function or at best checking whether hydronephrosis is relieved. This poses the possibility of overlooking the actual renal function of the stented kidney because serum creatinine or estimated glomerular filtration rate (eGFR) is usually normal due to compensatory action of the contralateral kidney even if the renal function of the stented kidney is deteriorated. This is especially true if stents are used unilaterally. Therefore, other means are necessary for renal function rather than just using laboratory values in order to evaluate the differential renal function of stented kidney.

Measuring kidney size may be a possible and practical mean for this purpose. Kidney size has long been used as an important parameter for clinical evaluation of kidney diseases. It is known to have significant correlation with kidney function [4]. It is also a determinant of renal prognosis [5]. Therefore, the objective of this study was to determine whether kidney sizes were changed after ureteral stents were instilled, and if so, what parameters were significant.

## 2. Materials and Methods

### 2.1. Study Population and Design

**Figure 1:**
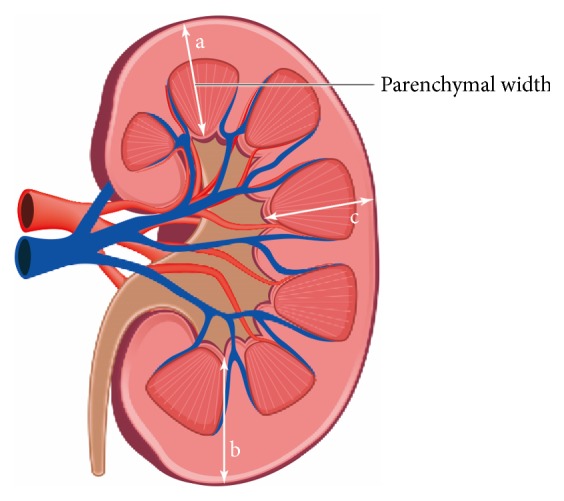
Parenchymal width (PW) of three points in the CT coronal view of kidney (upper, lower, and middle, designated as points a, b, and c, resp.) was measured. The mean PW of the three points was calculated.

The Institutional Review Board of St. Vincent's Hospital and Seoul St. Mary's Hospital, both of which are affiliations for the Catholic University of Korea, approved the study protocol. This was a bicenter retrospective study in which any patients from January 2010 to December 2015 who had indwelling unilateral ureteral stents were candidates. Polymeric ureteral stents were used for all patients. Inclusion criteria were as follows: first, patients with unilateral ureteral stents indwelled for more than 6 months; second, patients with abdominal computer tomography (CT) scans before ureteral stent placement and at the time of last stent change; third, patients with normal contralateral kidney. Exclusion criteria were as follows: first, patients with bilateral ureteral stents; second, patients with single kidney. Ureteral stents were changed every three months. Chart review was conducted to obtain the following information: age, sex, duration of ureteral stent indwelled (months), laterality of ureteral stent (left or right), underlying comorbidities (diabetes mellitus, hypertension, and chronic kidney disease), history of febrile urinary tract infection (UTI) episode that required admission, history of pelvic surgeries, history of pelvic radiation therapy (RT), history of chemotherapy, and underlying pathologic nature of ureteral obstruction (benign/malignant and internal/external). The estimated glomerular filtration rate (eGFR, mL/min/1.73 m^2^) was recorded before stenting and at the time of the last stent change. The % change of eGFR between the two points was calculated. eGFR was calculated using Modification of Diet in Renal Disease (MDRD) formula: eGFR (mL/min/1.73 m^2^) = 175 × (standardized *S*_cr_)^−1.154^  × (age)^−0.203^  × (1.212 if black) × (0.742 if female) [6]. To measure kidney size, all CT scans were reviewed and parenchymal width (PW) of three points in the coronal view of kidney (upper, lower, and middle, designated as points a, b, and c, resp.) was measured (Figure 1). PW was measured from the renal capsule to the renal collecting system. All measurements were done by a single investigator to reduce interobserver variation. The mean PW of the three points was calculated before stenting and at the time of the last stent change. The % change of the mean PW between the two points was calculated. The PW of the contralateral kidney was calculated using the same method.

### 2.2. Data Analysis

All data were analyzed using PASW (Predictive Analytics Software) Statistics for Macintosh, version 21.0 (SPSS Inc., Chicago, IL, USA). Descriptive statistics were used to describe baseline characteristics of the study population. Comparison of the mean PW and eGFR before stenting and at the time of the last stent change was done using either paired *t*-test or Wilcoxon signed rank test based on the result of Shapiro-Wilk test for normality. To find parameters related to the % change of mean PW, Spearman's rank correlation analysis and Mann–Whitney* U* test were performed. A *p* value of <0.05 was considered as statistically significant for all tests.

## 3. Results

Baseline characteristics of the study population are summarized in Table 1. The total number of eligible cases was 98. Mean age of the study population was 58.9 ± 10.9 (mean ± SD) years. The mean duration of ureteral stent indwelled was 15.6 ± 10.2 months. In 90.8% of these cases, malignancy was the cause of ureteral stent insertion. 43.9% of the patients had a history of pelvic RT. The reason for such high proportion of malignant causes was because only patients with CT scans available were included in this study; CT scans were not routinely used for patients with ureteral stents for benign causes.

**Figure 2:**
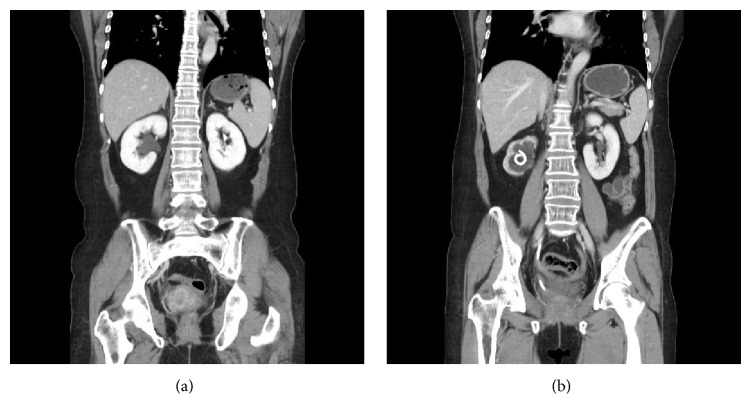
A CT scan of a patient with right hydronephrosis (a). Prominent shrinkage of renal parenchyma was noted (b) after 13 months of ureteral stent placement.

Changes of mean PW and eGFR before stenting and at the time of last stent change are shown in Table 2. The change of mean PW of stented kidneys was −16.9 ± 16.4 (mean ± SD)%, whereas the change of mean PW of unstented contralateral kidneys was 3.6 ± 10.7%. The difference of mean PW before stenting and at the time of the last stent change for both stented kidneys and contralateral kidneys was statistically significant (*p* < 0.05). To recapitulate, in a mean duration of 15.6 ± 10.2 months, PW of stented kidneys shrank an average of 17%, while unstented contralateral kidneys grew 3.6%, implying that contralateral kidneys compensated for the loss of renal function of stented kidneys. A representative CT scan of a patient showing this change of parenchymal width is shown in Figure 2. Moreover, eGFR before stenting and at the time of last stent change did not show significant difference (*p* = 0.294), indicating that eGFR did not reflect the decreased PW of stented kidneys. This again implied that compensation took place by contralateral kidneys.

**Figure 3:**
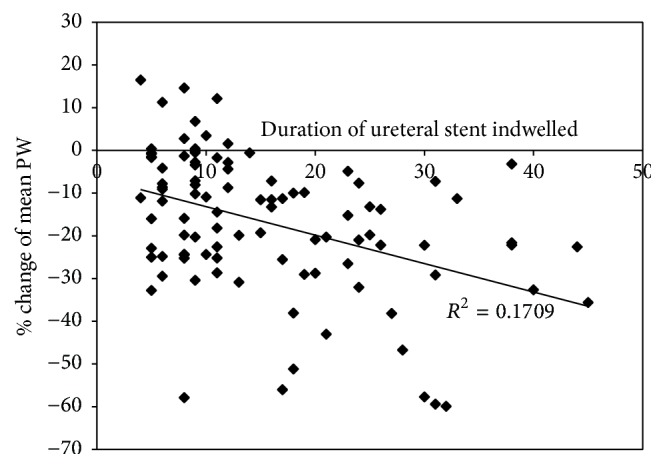
Scatter plot of % change of mean PW with regard to duration of ureteral stent indwelled showed a linear relationship with an *r*^2^ value of 0.1709. PW = parenchymal width.

To find out which parameters, if any, were related to the change of mean PW, Spearman's rank correlation analysis was performed for continuous variables and Mann–Whitney* U* test was conducted for categorical variables. Results are shown in Table 3. Based on correlation analysis, the % change of mean PW was found to be inversely related to the duration of ureteral stent indwelled (Spearman's correlation coefficient = −0.291, *p* < 0.001); the longer the ureteral stents indwelled, the smaller the kidneys became. Scatter plot of % change of mean PW with regard to duration of ureteral stent indwelled showed a linear relationship with an *r*^2^ value of 0.1709 (Figure 3). Based on Mann–Whitney* U* test, only history of chemotherapy among different parameters showed significant difference (*p* < 0.001); the kidney size shrank more in patients without history of chemotherapy. The % change of mean PW for patients without history of pelvic RT was −14.5 ± 13.2 (mean ± SD)%, whereas % change of mean PW for patients with history of pelvic RT was −19.9 ± 19.5%. The two % changes were not statistically significant (*p* = 0.190).

## 4. Discussion

It is a general consensus that, in cases of unilateral ureteral obstruction, ureteral stent can preserve renal function of the obstructed kidney unless ureteral stents fail for some reasons such as failure to relieve symptoms or hydronephrosis. As a result, clinicians may become somewhat indifferent to the selective function of individual stented kidney, especially when laboratory studies are normal. However, our observations of numerous patients with shrunken kidneys in spite of indwelling ureteral stent prompted us to question the efficacy of ureteral stents in preserving renal function of obstructed kidneys. Past reports have studied the efficacies of ureteral stents. However they were usually in the context of “stent failure.” The definitions for “stent failure” in most of these studies were failure to relieve symptoms or hydronephrosis, rise in creatinine, irritative symptoms due to ureteral stent, or the need for another procedure such as PCN [7–10]. To the best of our knowledge, no studies have focused on the efficacies of ureteral stents in terms of selective renal function of the stented kidneys.

In this study, measurement of PW was performed using CT scans to study the efficacy of unilateral ureteral stent in preserving selective renal function. Several reasons exist for this choice of methodology. First, conventional methods were inappropriate for this study. Measuring creatinine or eGFR is obviously improper for the purpose of this study because of the compensatory action of contralateral kidney [11]. Other methods for the determination of the individual renal function such as PCN and creatinine clearance or nuclear scintigraphy have limitations. PCN is too invasive while nuclear scintigraphy is often unreliable in cases of obstruction [12]. Second, CT is a widely used modality for evaluating obstructive uropathy of various etiologies. It not only provides excellent renal anatomy, but also is capable of calculating total and separate renal function, although sophisticated software might be needed to calculate total renal volume based on CT reconstructions [13]. Third, renal PW on CT scan appears to be able to predict relative renal function. In a study where renal parenchymal thickness ratio is compared to Mag-3 Lasix renogram [14], renal PW on CT scan was found to be significantly correlated to renogram function (correlation coefficient of 0.48, *p* < 0.001). Lastly, renal PW can be measured in a retrospective manner on existing CT scans with or without contrast. In addition, it does not need complex calculations or specific software for reconstruction [14]. Altogether, it can be reasoned that measuring PW on CT scans might be a simple and practical mean for studying selective renal function with ureteral stent.

The most important finding of this study was that, in cases of unilateral ureteral obstruction, renal PW was decreased over time in spite of indwelling ureteral stent, suggesting that ureteral stents might not be as efficacious in preserving renal function as most clinicians tend to believe. The current study also showed that the PW of contralateral unstented kidney was increased presumably due to compensation, and the resulting global renal function represented by eGFR was unchanged between before and after ureteral stent instilment.

The mechanism for the decrease in kidney size in spite of indwelling ureteral stent in the current study is currently unclear. However, several hypotheses can be made. First, impaired ureteral peristalsis resulting in inadequate urine drainage might be the reason. Ureteral peristalsis is needed for urine propulsion even with a stent in place [15]. The exact pathophysiological process is unclear. However, periureteral inflammatory reaction, edema [8], and ureteral smooth muscle dysfunction by external compression or malignancy [16, 17] may cause impairment of ureteral peristalsis. In fact, several studies have reported the high failure rate of ureteral stents in cases of ureteral obstruction caused by external compression or malignancy [7, 17–20]. The fact that the majority of the causes of obstruction was malignancy and external compression in the current study (both over 90%) suggests that impaired ureteral peristalsis might have caused dysfunction of ureteral stents, leading to decreased kidney size. Second, vesicoureteral reflux (VUR) might be another possible reason. Ureteral stents are capable of causing VUR [21]. Again, the exact mechanism is unclear. However, irritation of bladder mucosa caused by ureteral stent might induce spasm, subsequently increasing intravesical pressure; when coupled with the high vesical pressure during voiding, the reflux of bladder urine into the kidney can occur [22]. Since VUR is well known for its association with kidney atrophy and reduced relative renal function [23–25], VUR might have caused the decrease of kidney size in the current study.

Another possible reason that was expected to be significant was pelvic RT. RT can induce dose-related tissue damage including collagen deposition and endarteritis [26], resulting in chronic cystitis and bladder contracture in the bladder and ureteral strictures in ureters [27, 28]. This may lead to high bladder pressure and VUR, which will eventually damage the kidney. However, in the current study, although the kidneys of patients with history of pelvic RT shrank more than patients without such history (−19.9 ± 19.5 (mean ± SD)% versus −14.5 ± 13.2%), the two were not statistically different, contrary to our expectations. The small number of study population and failure to investigate specific information on RT such as dosage and location might be possible reasons for such negative result. Future studies are warranted in this respect.

One other interesting result of this study was for patients with history of chemotherapy. PW of patients without history of chemotherapy was decreased more than patients with history of chemotherapy, again contrary to our expectation. Our initial postulation was that kidney function would be compromised in patients with history of chemotherapy due to exposure to possible nephrotoxic drugs. The result shown in this study might be due to the heterogeneity of the study population; chemotherapy regimens, dosage, and type of causative malignancy were not considered. Again, the small number of study population limited us from performing further subgroup analysis based on chemotherapy regimen, dosage, and type of malignancy.

Several limitations exist in this study. First, the number of study population was relatively small. Second, the composition of the study population may not represent all patients with ureteral stents because the majority of the causes of ureteral obstruction in this study were from malignancy. Caution is needed when applying conclusions drawn from the current study to patients with ureteral obstruction due to benign causes. Third, information on bladder function, one of the possible causes of renal impairment, was lacking due to the retrospective nature of this study.

## 5. Conclusion

In cases of unilateral ureteral obstruction, kidney size was decreased over time despite indwelling ureteral stent, suggesting that ureteral stents might not be efficacious in preserving renal function. This finding can be overlooked by clinicians due to compensatory growth of the contralateral kidney and the resultant normal eGFR. Measuring PW in CT scans may be a simple and practical mean to study selective renal function with ureteral stent.

## Figures and Tables

**Table 1:** Study population.

*n*	98
Age (years), mean ± SD	58.9 ± 10.9
Duration of ureteral stent indwelled (months)	15.6 ± 10.2
Sex (female)	64/98 (65.3%)
DM	18/98 (18.4%)
HTN	30/98 (30.6%)
CKD	1/98 (1.0%)
Febrile UTI	9/98 (9.2%)
History of pelvic surgery	55/98 (56.1%)
History of pelvic RT	43/98 (43.9%)
History of chemotherapy	79/98 (80.6%)
Laterality (right)	50/98 (51.0%)
Malignancy	89/98 (90.8%)
Type of obstruction (external)	91/98 (92.9%)

SD = standard deviation, DM = diabetes mellitus, HTN = hypertension, CKD = chronic kidney disease, UTI = urinary tract infection, RT = radiation therapy.

**Table 2:** Changes of parenchymal width (stented and unstented contralateral kidney) and estimated glomerular filtration rate before and after indwelling ureteral stent.

	Before	After	% change	*p*
PW (stented kidney)	22.1 ± 3.5	18.5 ± 5.0	−16.9 ± 16.4	<0.001^1^
PW (contralateral kidney)	23.8 ± 4.3	24.6 ± 4.3	3.6 ± 10.7	0.003^2^
eGFR	82.8 ± 27.1	78.9 ± 26.1	−0.18 ± 32.1	0.294^1^

Mean duration: 15.6 ± 10.2 months; ^1^Wilcoxon signed ranks test; ^2^paired *t*-test; PW = parenchymal width, eGFR = estimated glomerular filtration rate.

**Table 3:** Analysis of continuous and categorical variables.

	% change of mean PW	*p*
Continuous variables	Spearman's correlation coefficient	
Age	−0.067	0.336^1^
Duration	−0.291	<0.001^1^
Categorical variables	mean ± SD	
Sex		0.185^2^
Male	−13.3 ± 15.4	
Female	−18.8 ± 16.7	
DM		0.401^2^
No	−16.4 ± 16.8	
Yes	−19.1 ± 14.7	
HTN		0.694^2^
No	−16.0 ± 15.9	
Yes	−19.1 ± 17.4	
History of pelvic surgery		0.657^2^
No	−15.9 ± 15.2	
Yes	−17.7 ± 17.3	
History of pelvic RT		0.190^2^
No	−14.5 ± 13.2	
Yes	−19.9 ± 19.5	
History of chemotherapy		<0.001^2^
No	−28.0 ± 14.5	
Yes	−14.2 ± 15.7	
Laterality		0.304^2^
Left	−18.0 ± 15.8	
Right	−15.8 ± 17.0	

^1^Spearman's rank correlation analysis; ^2^Mann–Whitney *U* test; PW = parenchymal width, eGFR = estimated glomerular filtration rate, SD = standard deviation, DM = diabetes mellitus, HTN = hypertension, RT = radiotherapy.
